# Effectiveness and safety of normoxic allogenic umbilical cord mesenchymal stem cells administered as adjunctive treatment in patients with severe COVID-19

**DOI:** 10.1038/s41598-023-39268-2

**Published:** 2023-08-02

**Authors:** Bintang Soetjahjo, Rusdy Ghazali Malueka, Arief Nurudhin, Purwoko, Sumardi, Rudi Wisaksana, Artrien Adhiputri, Sudadi, Arto Yuwono Soeroto, Brigitte Rina Aninda Sidharta, Jarir At Thobari, Tri Wahyu Murni, Widiastuti Soewondo, Elizabeth Henny Herningtyas, Reza Widianto Sudjud, Ika Trisnawati, Nur Rahmi Ananda, Ahmad Faried

**Affiliations:** 1 Department of Orthopaedics and Traumatology, Universitas Sebelas Maret-Dr. Moewardi Hospital, Solo, Indonesia; 2 Department of Neurology, Faculty of Medicine, Public Health and Nursing, Universitas Gadjah Mada-Dr. Sardjito General Hospital, Yogyakarta, Indonesia; 3 Department of Internal Medicine, Universitas Sebelas Maret-Dr. Moewardi Hospital, Solo, Indonesia; 4 Department of Anesthesiology and Intensive Therapy, Universitas Sebelas Maret-Dr. Moewardi Hospital, Solo, Indonesia; 5 Department of Internal Medicine, Faculty of Medicine, Public Health and Nursing, Universitas Gadjah Mada-Dr. Sardjito General Hospital, Yogyakarta, Indonesia; 6 Department of Internal Medicine, Universitas Padjadjaran-Dr. Hasan Sadikin Hospital, Bandung, Indonesia; 7 Department of Pulmonology and Respiratory Medicine, Universitas Sebelas Maret-Dr. Moewardi Hospital, Solo, Indonesia; 8 Department of Anesthesiology and Intensive Therapy, Faculty of Medicine, Public Health and Nursing, Universitas Gadjah Mada-Dr. Sardjito General Hospital, Yogyakarta, Indonesia; 9 Department of Clinical Pathology, Universitas Sebelas Maret-Dr. Moewardi Hospital, Solo, Indonesia; 10 Department of Pharmacology and Therapy, Faculty of Medicine, Public Health and Nursing, Universitas Gadjah Mada, Yogyakarta, Indonesia; 11 Department of Surgery, Universitas Padjadjaran-Dr. Hasan Sadikin Hospital, Bandung, Indonesia; 12 Department of Radiology, Universitas Sebelas Maret-Dr. Moewardi Hospital, Solo, Indonesia; 13 Department of Clinical Pathology, Faculty of Medicine, Public Health and Nursing, Universitas Gadjah Mada-Dr. Sardjito General Hospital, Yogyakarta, Indonesia; 14 Department of Anesthesiology-Intensive Therapy, Universitas Padjadjaran-Dr. Hasan Sadikin Hospital, Bandung, Indonesia; 15 Department of Neurosurgery, Universitas Padjadjaran - Dr. Hasan Sadikin Hospital, Bandung, 40161 Indonesia

**Keywords:** Biochemistry, Biotechnology, Cell biology, Drug discovery, Genetics, Immunology, Microbiology, Molecular biology, Neuroscience, Stem cells, Diseases

## Abstract

Inflammatory response in COVID-19 contributes greatly to disease severity. Mesenchymal Stem Cells (MSCs) have the potential to alleviate inflammation and reduce mortality and length of stay in COVID-19 patients. We investigated the safety and effectiveness of normoxic-allogenic umbilical cord (NA-UC)-MSCs as an adjunctive treatment in severe COVID-19 patients. A double-blind, multicentric, randomized, placebo-controlled trial involving severe COVID-19 patients was performed from January to June 2021 in three major hospitals across Java, Indonesia. Eligible participants (n = 42) were randomly assigned to two groups (1:1), namely the intervention (n = 21) and control (n = 21) groups. UC-MSCs dose was 1 × 10^6^ /kg body weight on day D0, D3, and D6. The primary outcome was the duration of hospitalization. Meanwhile, the secondary outcomes were radiographical progression (Brixia score), respiratory and oxygenation parameters, and inflammatory markers, in addition to the safety profile of NA-UC-MSCs. NA-UC-MSCs administration did not affect the length of hospital stay of severe COVID-19 patients, nor did it improve the Brixia score or mMRC dyspnoea scale better than placebo. Nevertheless, NA-UC-MSCs led to a better recuperation in oxygenation index (120.80 ± 72.70 baseline vs. 309.63 ± 319.30 D + 22, *p* = 0.038) and oxygen saturation (97.24 ± 4.10% vs. 96.19 ± 3.75% in placebo, *p* = 0.028). Additionally, compared to the placebo group, the treatment group had a significantly smaller increase in PCT level at D + 22 (1.43 vs. 12.76, *p* = 0.011). No adverse effects, including serious ones, were recorded until D + 91. NA-UC-MSCs therapy is a very safe adjunct for COVID-19 patients. It improves the oxygenation profile and carries potential to suppress inflammation.

## Introduction

The emergence of the Coronavirus disease 2019 (COVID-19) pandemic has highlighted the need for streamlined drug development and validation within a condensed timeframe^1,2^. While most COVID-19 patients do not require hospitalization, the frequent outbreak of severe cases and the introduction of new variants compromise the availability of hospital beds and may aggravate mortality. These patients often present with acute respiratory distress syndrome (ARDS), which ultimately deteriorates into multiple organ failure^3^. The latter may be triggered by the attachment of the SARS-CoV-2 virus on the receptors of target organs and also by a cytokine storm response^4,5^.

The severity of the disease is not determined by the SARS-CoV-2 viral load but rather by the inflammatory response, which may be quantified in plasma samples^6,7^. The abnormally elevated pro-inflammatory and anti-inflammatory cytokines in severe COVID-19 patients, dubbed the "cytokine storm", suggested that these patients have a dysfunctional immune system^7,8^. These comprise, but are not limited to, VEGF, TNF-α, SCF, LIF, IL-2, IL-4, IL-6, IL-8, IL-10, IL-15, IL-17A, IL-18, IL-1β, and IFN-γ^9^. The cytokine storm subsequently leads to the cytokine release syndrome (CRS), which, together with chemokine release, forms the basis for multiple organ failure^4,10^.

As no approved pharmaceuticals have been demonstrated to reduce viral load, all therapy in COVID-19 is directed towards improving symptoms and immune response^11–13^. Hence, the discovery and investigation of various compounds and biologicals (*e.g., cells*) have been carried out, partly propelled by ongoing confusion and desperation. Mesenchymal stem cells (MSCs), in particular, is a biological with potent angiogenetic, anti-apoptotic, anti-inflammatory, and immunomodulatory properties, all of which are beneficial for the recovery of COVID-19 patients^14,15^. Multiple clinical trials have discovered that MSCs therapy reduced mortality and promoted recovery in severe COVID-19, a finding strengthened by a recent meta-analysis^16,17^. For example, one Indonesian trial found that IL-6, a pro-inflammatory cytokine, and ferritin levels were decreased in MSCs-administered patients^14^. In another, PaO_2_/FiO_2_ ratio and radiological profile in severe COVID-19 patients were drastically improved after umbilical cord (UC)-MSCs therapy^15^.

Besides these potentials, one notable aspect of allogenic MSCs application is its economic efficiency. Firstly, they are prepared by extracting cord blood or cutting up Wharton's Jelly (WJ), readily available in virtually all hospitals with maternity wards, and culturing them inside flasks^18^. They can be passaged for 12–13 cycles before growth deceleration is observed^19^. Secondly, unlike their secretome derivatives, UC-MSCs require less sophisticated equipment to process; its collection is non-invasive, abundant sources, immunomodulatory properties, low risk of rejection^14,20^. Regarding the dose given, we carefully selected based on several clinical trial and decided to use the UC-MSCs with dose 1 × 10^6^/kg body weight on day D0, D3, and D6 (ClinicalTrials.gov ID: NCT04333368). Based on this, we investigated the safety and effectiveness of our normoxic-allogenic (NA)-UC-MSCs (the quality control the product see supplement 1) as an adjunctive treatment in severe COVID-19 patients.

## Results

### Recruitment

**Figure 1:**
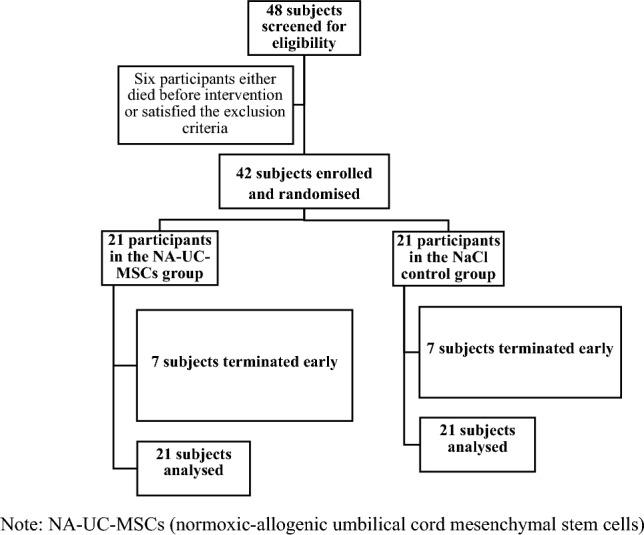
Participant recruitment process. *Note*: NA-UC-MSCs (normoxic-allogenic umbilical cord mesenchymal stem cells).

Recruitment patients using both the Indonesian Ministry of Health guidelines on COVID-19 and Wu Z, McGoogan JM criteria were consulted for severe COVID-19 categorisation^21,22^. Severe COVID-19 was defined as COVID-19 pneumonia in addition to at least one of the following: SpO_2_ ≤ 93% on room air, PaO_2_/FiO_2_ ≤ 300 mmHg, or a respiratory rate ≥ 30 breaths/ min. Forty-eight subjects were recruited (from 48 screen candidates) from 30 Jan to 24 Jun 2021. The screening was performed in the three aforementioned hospitals across Java, Indonesia. Six subjects were excluded from the study due to either having been deceased before the commencement of the trial or having satisfied the exclusion criteria. In total, 42 eligible subjects were randomized into two equal groups: 21 in the intervention group and 21 in the placebo control group. This is a double blind randomized study organized by sponsor, Contract Research Organization (CRO). Randomization was carried out using the block size 4 method for the two test groups with a total of 42 numbers prepared by the CRO. The collection of blood samples and other parameters was performed in accordance with the study methods. A total of 14 patients, seven in each group, died before the end of the observation period (see detailed in supplement 2). The detailed process of subject recruitment is displayed in Fig. 1.

### General subject characteristics

The general comparison between the NA-UC-MSCs and the placebo groups was summarised in Table 1. There were no significant disparities in most baseline characteristics of subjects between the two groups. The average age of participants in the intervention and control groups were 56.10 and 55.86 years, respectively. The average body weight were 68.05 and 68.33 in the intervention and control groups, respectively. In addition, subjects were also evenly stratified by gender across the two groups. No difference was observed in past medical history, as indicated by diabetes, hypertension, chronic kidney diseases, congestive heart failures (CHF), chronic liver diseases, chronic obstructive pulmonary disease (COPD), stroke, autoimmune diseases, and active smoking status.

**Table 1:** General Characteristics of Participants.

Characteristic	NA-UC-MSCs group (n = 21)	Control group (n = 21)	*p*-value
Age			
Mean	56.10 ± 12.49	55.86 ± 10.17	0.668
Minimum	24	32	
Maximum	71	71	
Sex			
Female	11 (52.4%)	9 (42.9%)	0.542
Male	10 (47.6%)	12 (57.1%)	
Body weight (kg)			
Mean ± SD	68.05 ± 12.78	68.33 ± 15.97	0.850
Minimum	50	50	
Maximum	92	120	
Medical history			
Diabetes			
Yes	8 (38.1%)	9 (42.9%)	1.000
No	13 (61.9%)	11 (52.4%)	
Unknown	0	1 (4.8%)	
Hypertension			
Yes	9 (42.9%)	10 (47.6%)	1.000
No	12 (57.1%)	9 (42.9%)	
Unknown	0	2 (9.5%)	
Chronic kidney disease			
Yes	3 (14.3%)	0	0.983
No	18 (85.7%)	20 (95.2%)	
Unknown	0	1 (4.8%)	
CHF			
Yes	3 (14.3%)	0	0.983
No	18 (85.7%)	20 (95.2%)	
Unknown	0	1 (4.8%)	
Chronic liver disease			
Yes	1 (4.8%)	0	1.000
No	20 (95.2%)	20 (95.2%)	
Unknown	0	1 (4.8%)	
COPD			
Yes	0	1 (4.8%)	1.000
No	21 (100%)	19 (90.5)	
Unknown	0	1 (4.8%)	
Stroke			
Yes	0	2 (9.5%)	1.000
No	21 (100%)	18 (85.7%)	
Unknown	0	1 (4.8%)	
Autoimmune disease			
Yes	0	0	1.000
No	21 (100%)	20 (95.2%)	
Unknown	0	1 (4.8%)	
Active smoker			
Yes	6 (28.6%)	3 (15%)	0.992
No	15 (71.4%)	17 (85%)	
Unknown	0	1	

Note: NA-UC-MSCs (normoxic-allogenic umbilical cord mesenchymal stem cells).

### Duration of hospitalisation

The average hospital stay in subjects who received NA-UC-MSCs was 20.81 ± 12.25 days compared to control subjects, who were treated for 16.81 ± 5.63 days as shown in Table 2. No significant difference was observed in the length of hospitalization between the two groups.

**Table 2:** Duration of Hospitalisation (Days) Since Admission.

Group	Mean ± S.D	Median-mode	Min–max	*p
NA-UC-MSC (n = 21)	20.81 ± 12.25	20–20	6–57	0.427
Control (n = 21)	16.81 ± 5.63	17–20	6–27	

* Normal distribution, *p*-value calculated using Independent T-Test.

### Radiographical severity (Brixia score)

**Figure 2:**
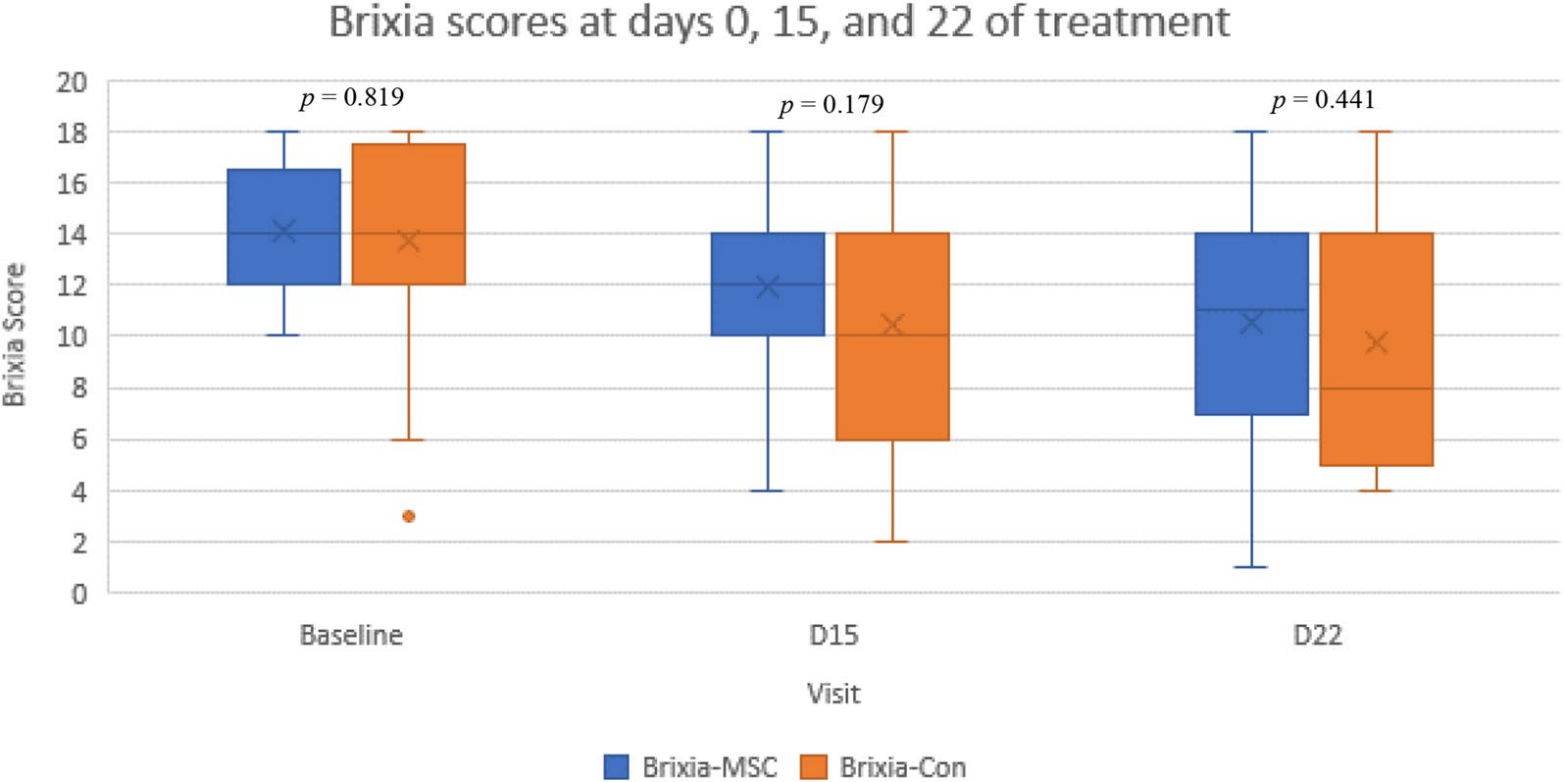
Mean Brixia scores at days 0, 15, and 22 of treatment.

Table 3 and Fig. 2 portray the subjects’ mean Brixia scores across both groups and time points. Brixia scores were assessed by evaluating the subjects’ chest x-ray examination on days 0, 15, and 22 of intervention. Although no significant difference between groups was apparent, NA-UC-MSCs successfully alleviated the Brixia score on day 15 and the subsequent day 22.

**Table 3:** Brixia score between groups.

	Baseline	D15	D22	**p
NA-UC-MSCs	14.00 (12.00–16.50)	12.00 (10.00–14.00)	11.00 (7.00–14.00)	0.016
Control	14.00 (12.00–17.50)	10.00 (6.00–14.00)	8.00 (5.00–14.00)	0.009
*p	0.819	0.179	0.441	

Variables were presented as median (IQR). *p-value calculated using Mann–Whitney’s test. *p < 0.05 shows significant differences between MSC and control group. **p-value calculated using Friedman’s test. **p < 0.05 shows significant differences between time point each group. If *p* is significant, posthoc test entail (supplement). *IQR* Interquantile range, *MSCs*: Mesenchymal stem cells group, *CON* Control NaCl group.

### MMRC dyspnoea scale, PEF, and 6 MW test

**Figure 3:**
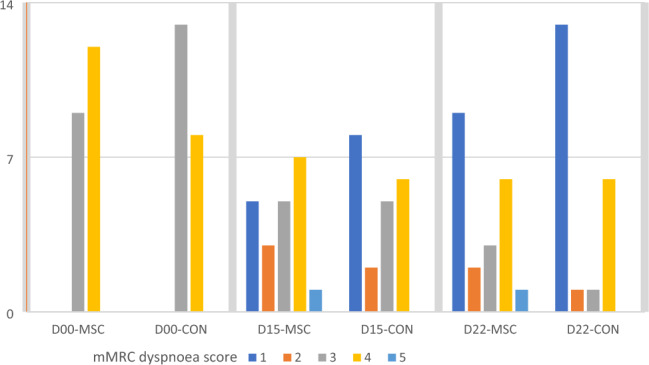
MMRC dyspnoea scale in the intervention and control groups.

**Figure 4:**
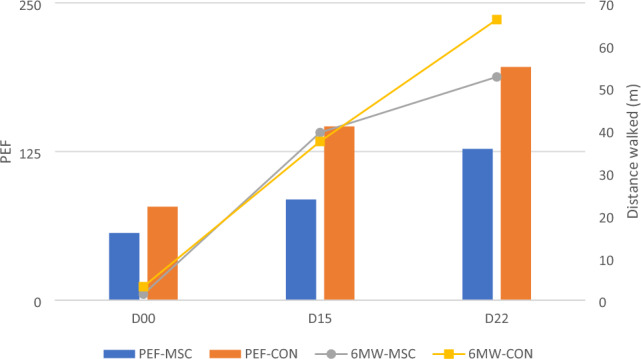
PEF and 6 MW distance across both treatment groups.

Table 4 and Fig. 3 present the mMRC dyspnoea scores, while Table 5 and Fig. 4 elaborate on the PEF and 6 MW test results. Both the NA-UC-MSCs and the control group showed improvement in dyspnoea scores on days 15 and 22 after treatment. However, MSCs administration did not seem to relieve or aggravate dyspnoea scores compared to control. A similar pattern is observed in the 6 MW test, where no inter-group difference was found at any point in time.

**Table 4:** MMRC dyspnoea scale over time across groups.

	Baseline	D + 15	D + 22	**p
	n (%)	n (%)	n (%)	
NA-UC-MSCs group				
Score 1		5 (11.9%)	9 (21.4%)	< 0.001
Score 2		3 (7.1%)	2 (4.8%)	
Score 3	9 (21.4%)	5 (11.9%)	3 (7.1%)	
Score 4	12 (28.6%)	7 (16.7%)	6 (14.3%)	
Score 5		1 (2.4%)	1 (2.4%)	
Control				
Score 1		8 (19.0%)	13 (31.0%)	< 0.001
Score 2		2 (4.8%)	1 (2.4%)	
Score 3	13 (31.0%)	5 (11.9%)	1 (2.4%)	
Score 4	8 (19.0%)	6 (14.3%)	6 (14.3%)	
Score 5				
*p	0.222	0.347	0.301	

Note: NA-UC-MSCs (normoxic-allogenic umbilical cord mesenchymal stem cells).

* *p*-value calculated using Mann–Whitney’s test.

**Table 5:** PEF and 6 MW distance (m) between two groups.

		Baseline	D15	D22	**p
PEF	MSCs	0.00 (0.00–135.37)	0.00 (0.00–192.50)	60.00 (0.00–275.00)	0.154
	CON	30.00 (0.00–155.00)	100.00 (0.00–225.00)	165.00 (45.00–305.00)	**0.026**
*p		0.508	0.333	0.268	
Walk distance (m)	6 MW	0.00 (0.00–3.00)	0.00 (0.00–6.00)	0.00 (0.00–6.00)	0.142
	6 MW	0.00 (0.00–0.00)	0.00 (0.00–27.00)	0.00 (0.00–72.00)	**0.001**
*p		0.556	0.631	0.630	

Variables were presented as median (IQR). *p-value calculated using Mann–Whitney’s test. *p < 0.05 shows significant differences between MSC and control group. **p-value calculated using Friedman’s test. **p < 0.05 shows significant differences between time point each group. If *p* is significant, posthoc test entail (supplement). IQR: Interquantile range; PEF: Peak Expiratory Flow; 6 MW: 6 Min Walk; MSCs: Mesenchymal Stem Cells Group; CON: Control NaCl Group.

Significant values are in [bold].

### ***Oxygenation index, duration of oxygenation, and O***_***2***_*** saturation***

**Figure 5:**
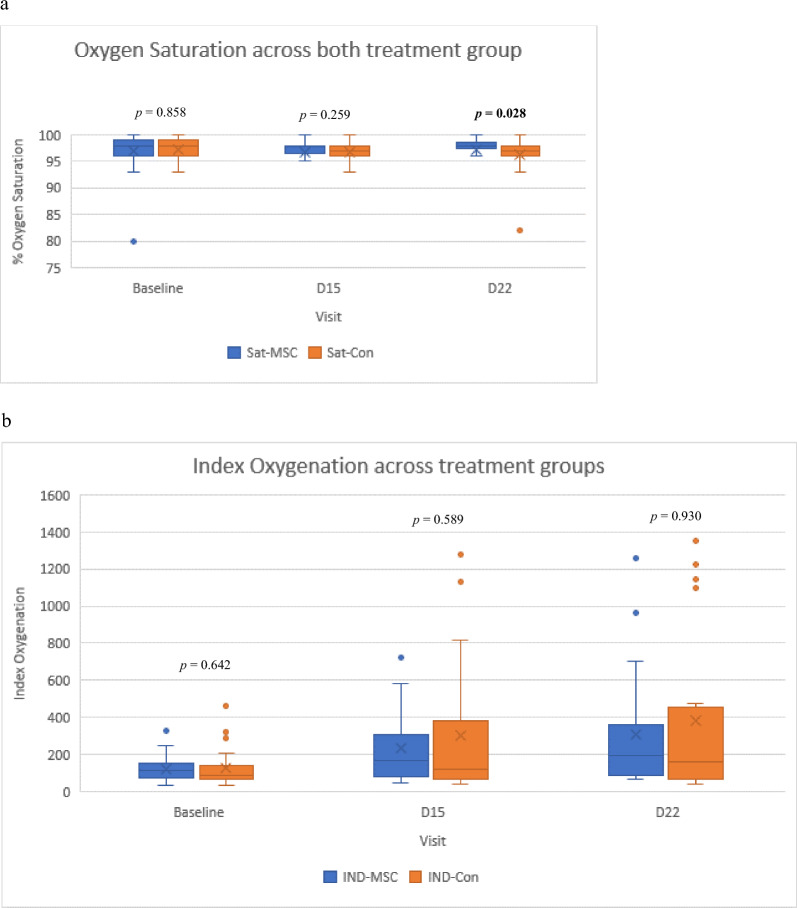
Oxygenation index and oxygen saturation across both treatment groups.

Table 6 and Fig. 5 depict the oxygenation index, duration of oxygenation, and oxygen saturation across both groups at different time points. Overall, it can be inferred that NA-UC-MSCs significantly improved the oxygenation index. This was not observed in the control group. Intriguingly, oxygen saturation by day 22 also favoured the intervention group.

**Table 6:** Oxygen index, duration oxygenation, and O2 saturation across group.

		Baseline	D15	D22 MSC	**p
Index oxygenation	MSCs	114.46 (74.22–156.40)	166.80 (83.84–310.93)	191.50 (84.69–364.05)	**0.038**
	CON	84.70 (65.35–143.33)	120.10 (65.35–384.76)	162.00 (69.18–453.09)	0.214
		0.642	0.589	0.930	
Duration of O_2_ administration	MSCs		13 (61.9%)	8 (38.1%)	
	CON		13 (61.9%)	8 (38.1%)	
***p		1.000			
Oxygen saturation	MSCs	98.00 (96.00–99.00)	98.00 (96.50–98.00)	98.00 (97.50–98.50)	0.313
	CON	98.00 (96.00–99.00)	97.00 (96.00–98.00)	97.00 (96.00–98.00)	0.640
*p		0.858	0.259	**0.028**	

Oxygen index, and O_2_ saturation were presented as median (IQR). *p-value calculated using Mann–Whitney’s test. *p < 0.05 shows significant differences between MSC and control group. **p-value calculated using Friedman’s test. **p < 0.05 shows significant differences between time point each group. If *p* is significant, posthoc test entail (supplement). *** Defined as oxygen therapy having been terminated by Dth day of treatment. IQR: Interquantile range; MSCs: Mesenchymal Stem Cells Group; CON: Control NaCl Group.

Significant values are in [bold].

### PCT, ESR, and CRP markers

**Figure 6:**
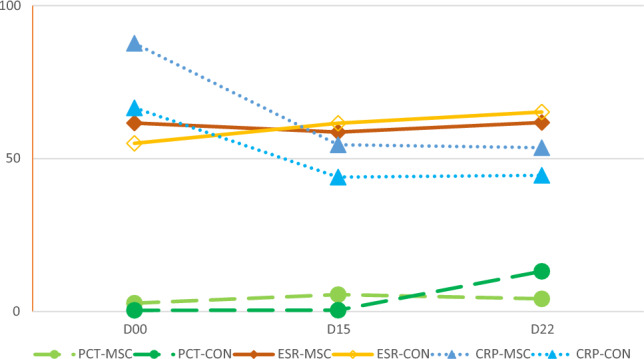
Evolution of PCT, ESR, and CRP levels on days 15 and 22 post-intervention. A marked reduction in CRP levels was seen in both groups.

Table 7 and Fig. 6 demonstrate that NA-UC-MSCs administration did not alter PCT, ESR, and CRP levels compared to placebo. Despite this, Fig. 6 evolution of PCT, ESR, and CRP levels on days 15 and 22 post-intervention. A marked reduction in CRP levels was seen in both groups. We then performed an additional analysis by comparing the difference in PCT values between baseline and day 22 in the two groups. As shown in Table 8, the treatment group had a significantly smaller increase in PCT value than the control group (mean changes 1.43 vs. 12.76, *p* = 0.011).

**Table 7:** PCT, ESR and CRP level across groups.

		Baseline	D15	D22	**p
PCT	MSCs	0.17 (0.50–0.43)	0.11 (0.05–0.30)	0.09 (0.05–0.51)	0.269
	CON	0.09 (0.05–0.12)	0.09 (0.05–0.26)	0.23 (0.09–0.68)	0.062
*p		0.184	0.919	0.186	
ESR	MSCs	65.00 (33.00–82.00)	57.00 (31.00–85.50)	57.00 (40.00–81.50)	0.229
	CON	52.00 (29.50–87.00)	72.00 (44.50–85.00)	74.00 (41.50–84.00)	0.683
*p		0.554	0.715	0.497	
CRP	MSCs	96.40 (20.95–142.50)	9.20 (5.00–108.50)	22.70 (3.90–98.50)	**0.046**
	CON	79.00 (16.00–97.00)	23.50 (8.10–76.75)	14.90 (2.60–86.00)	**0.001**
*p		0.273	0.880	0.642	

Variables were presented as median (IQR). *p-value calculated using Mann–Whitney’s test. *p < 0.05 shows significant differences between MSC and control group. **p-value calculated using Friedman’s test. **p < 0.05 shows significant differences between time point each group. If *p* is significant, posthoc test entail (supplement). *IQR* Interquantile range, *PCT* Procalcitonin, *ESR* Erythrocyte sedimentation rate, CRP C-reactive protein, *MSCs* Mesenchymal stem cells group, *CON* Control NaCl group.

Significant values are in [bold].

**Table 8:** Differences in Mean Changes in PCT between groups.

Group	Time	Means ± S.D	Mean changes (Δ)	*p
MSCs	Baseline	2.74 ± 10.59	2.81	0.236
	D + 15	5.55 ± 17.18		
Con	Baseline	0.41 ± 1.30	0.05	
	D + 15	0.46 ± 1.30		
MSCs	Baseline	2.74 ± 10.59	1.43	**0.011**
	D + 22	4.17 ± 12.38		
Con	Baseline	0.41 ± 1.30	12.76	
	D + 22	13.17 ± 57.17		

Note: MSCs (mesenchymal stem cells), Con (control).

Significant values are in [bold].

### Survival

A total of 14 subjects (33.3%) were deceased during the course of therapy. Among those who died, seven (16.7%) were in the NA-UC-MSCs group, and the other seven (16.7%) were from the control group. These subjects were then classified as "early terminated" patients.

## Discussion

The intravenous administration of NA-UC-MSCs did not affect the duration of hospitalization in severe COVID-19 patients. This study, however, confirmed the safety of NA-UC-MSCs therapy in these subjects. We choose the duration of stay in the hospital as the primary outcome since we want to clarify previously study that was found the length of hospitalization and ICU stays were shorter in the experimental group, when compared to the control group. However, most of the differences were not statistically significant^23,24^. Therefore, further studies involving UC-MSCs should also incorporate the length of intensive care rather than gross hospital stay per se.

The Brixia score is a new, potent inventory in determining the current condition and prognosis of pneumonia in COVID-19 patients. It was invented by Borghesi et al.^25,26^ and has subsequently been adapted worldwide. In our study, NA-UC-MSCs were found to relieve Brixia scores in a similar manner to the NaCl placebo. A similar finding was discovered by Abdullah et al.^27^, where MSCs secretomes were administered instead. The similarity between the two groups suggested that NA-UC-MSCs therapy is a safe approach for adjunctive treatment. However, the delivery of MSCs or secretomes to the target lung tissues may be compromised.^28^.

Oxygen saturation, dyspnoea, and oxygen therapy are crucial inventories in managing COVID-19 patients. The former and the middle has been entrenched as an indicator of a patient’s survival^29^, while the latter is a relatively simple, heuristic aid that acts as a countermeasure to address the problem^30^. In our study, we failed to show that NA-UC-MSCs therapy influenced mMRC dyspnoea scale progression nor shortened the length of oxygenation, which was consistent with findings by Shi et al. (2021), who suggested that UC-MSCs administration would only be beneficial to improve these parameters when administered during the acute progressive stage of SARS-CoV-2 infection^31^. Interestingly, peripheral oxygen saturation favoured the MSCs group by day 22 (97.24% vs. 96.19% in controls), and additionally, the oxygenation index (PaO_2_/FiO_2_) skyrocketed by 156% the baseline value by day 22 in the intervention group. The latter replicates the results of a trial by Iglesias et al. (2021), whose five participants had their oxygenation index improved from 76 to 154 within a week upon NA-UC-MSCs treatment.^32^ However, as to how a more downstream parameter is influenced by bypassing the scope of a more upstream variable (dyspnoea and oxygen therapy) is subject to a more profound investigation. Moreover, a more distal variable, the distance travelled for six Min, did not differ in both groups across the two assessment points.

We also tracked the levels of three key inflammatory markers: PCT, ESR, and CRP. Out of the three, only CRP showed a significant reduction in both groups. This suggested no difference in bacterial co-infection between groups since PCT is a powerful biomarker for inflammation of bacterial origin^33^. While no significant differences between groups were observed, we managed to suppress CRP levels in the MSCs and control groups (53.57 mg/mL and 44.54 mg/mL) after 22 days, which ultimately translates to the sufficient safety of MSCs. As a comparison, a study by Karyana et al.^34^ in nine patients yielded a median 28 day CRP of 0.45 mg/L in the MSCs group, compared to 1.1 mg/L in the placebo group. However, the MSCs used were of embryonic origin, dubbed “DW-MSCs”, whose procurement and logistics would involve extra steps; and the participants had low-risk COVID-19.

Additionally, we found that on the day 22 of the intervention, the treatment group had a significantly smaller increase in PCT value compared to the control group. Procalcitonin is a commonly used diagnostic marker in bacterial infection. However, previous studies showed that in COVID-19, procalcitonin is a biomarker for disease severity rather than bacterial co-infection. High procalcitonin level has been shown to be associated with disease severity, increased intensive unit admission, and mortality in patients with COVID-19. A decrease in PCT level has also been shown as an indication of recovery from COVID-19^35,36^. Our results raise the possibility that the administration of NA-UC-MSCs could probably prevent more severe progression of COVID-19. This, however, warrants further study.

Clinically, COVID-19 cases may be classified as asymptomatic, mild, moderate, or severe with respiratory failure, with the lattermost frequently leading to death^37^. In our trial, 14 (33.33%) deaths during the study period were reported, which were evenly stratified across the NA-UC-MSCs group (7, 16.67%) and the control group (7, 16.67%). A recent report suggested that this death rate exceeded that in the US, which were 12.7% for oxygenated patients and 47.5% for mechanically-ventilated patients^38^. Nevertheless, it is still comparable to another Indonesian investigation of MSCs in severe COVID-19 patients, where 25% of deaths occurred in the MSCs group compared to 45% in the control group^14^. To reinforce this, the Wharton’s jelly MSCs COVID-19 study noted a 30% death rate in the MSCs group and 60% in the control group^23^.

The two primary limitations of our study were the relatively small number of participants and the absence of a window period to recruit them. A greater number of subjects would allow a finer distinction between truly significant associations and those that occurred by chance. Complementarily, due to the hospitals' hierarchical status being the highest in each respective region, potential eligible subjects may be screened before receiving referral patients. If a window period had been properly defined (i.e., days 0–3 since positive PCR confirmation) and implemented, more pronounced response to MSCs therapy can be expected^31^.

The two other crucial limitations were the choice of inflammatory markers and a single delivery route. In numerous other studies, cytokines (e.g., IL-6, TNF-α) and VEGF were quantified in addition to ESR and CRP^16,17,39,40^. Doing so would allow the authors to infer more direct causation and mechanism of MSCs therapy in improving both the respiratory and general condition of COVID-19 patients. For example, an elevated IL-6 is putatively triggered as a response to injury in the alveolar epithelial cells^6^, while a high VEGF may indicate a critical illness^9^.

The route of MSCs delivery may also be varied to better quantify the patient's response to each. It has been established that intramuscular or intra-organ delivery of MSCs provides a more robust response compared to the systemic intravenous route^28^. Since the lung parenchyma is an anatomically inconvenient location for local injection, we would suggest an upcoming investigation on inhalational or intrabronchial MSCs. These two techniques transcend the limits of intravenous delivery and have been safely implemented for COVID-19 patients^41^. While our study did not note any adverse effects or increased mortality and is, therefore, safe, we acknowledge that imprudent use of MSCs may harm patients by promoting pro-coagulant AE, which manifests in the form of thrombo-embolic ischemia or disseminated intravascular coagulation^28^.

## Methodology

### Study design

This study was a multicentric, double-blinded, randomized, placebo-controlled trial to evaluate NA-UC-MSCs as a complementary treatment in severe COVID-19 patients (clinicaltrials.gov registration number NCT05132972 24/11/2021). This study approved by Health Research Ethics Committee, National Institute of Health Research and Development (HREC-NIHRD); ethical clearance number LB.02.01/2/KE.573/2020 extended no. LB.02.01/2/KE.582/2021. All experiments were conducted in accordance with the relevant guidelines and regulations.

### Participants

Subjects were picked via stratified random sampling, then randomized via a computerized random number generator. The total number of subjects was 42, divided into 21 subjects in the NA-UC-MSCs group and 21 in the placebo (NaCl) group. They hail from three major hospitals across the island of Java: Dr. Hasan Sadikin General Hospital in Bandung, Dr. Moewardi General Hospital in Surakarta, and Dr. Sardjito General Hospital in Yogyakarta. Recruitment was done from 30 January to 24 June 2021.

All subjects who participated in this trial had satisfied the inclusion and exclusion criteria. The inclusion criteria were: (1) Subjects 18–75 years old, who had been positively diagnosed with COVID-19 based on real-time reverse-transcriptase polymerase chain reaction (RT-PCR) assay of either pharyngeal, sputum, or broncho-alveolar lavage swab specimen; (2) Subjects are classified as severe COVID-19 patients; (3) Not mechanically ventilated upon admission; (4) No other adjunctive treatments were administered; (5) Agreed to participate and signed the informed consent. Meanwhile, the exclusion criteria were: (1) Pregnant, lactating women, or women on a contraceptive program; (2) a history or diagnosis of tumors or a history of breast cancer or ovarian cancer in the mother or sister; (3) SGPT/ALT value five times the upper limit of the normal value; (4) eGFR value < 30 mL/min; (5) requires invasive ventilation; (6) shock; (7) complications of organ failure; (8) had enrolled in other clinical trials within the last 3 months.

### Randomization and masking

All eligible subjects (n = 42) were randomly assigned into two groups (1:1 ratio) to either the intervention group, who were to receive NA-UC-MSCs (n = 21), or the placebo group, who were administered with NaCl (n = 21). Randomization was performed using blocks (block size = 4), and subjects were assigned to both groups with randomization software (sealedenvelope.com) by an independent statistician. The trial was prepared by a third party with no responsibility for patient care and data collection. All subjects, investigators, and treating physicians were blinded.

### NA-UC-MSCs preparation

Human umbilical cords were obtained from the cesarean section in the maternity ward of a hospital in Jakarta from a qualified donor that passed donor screening and testing of infectious disease (HIV, Hepatitis B, Hepatitis C, Syphilis, CMV IgM) and karyotyping. The human umbilical cord was subsequently processed to yield MSCs in Regenic Laboratory PT. Bifarma Adiluhung, Jakarta, Indonesia, with GMP standard. The MSCs were isolated with Alpha MEM (+ GlutaMAX) + Human Serum + Growth Factors and Vitamin + Antibiotic Antimicotic 1% and then passaged to passage 7 with Alpha MEM (+ GlutaMAX) + Human Platelet Lysate. To positively identify MSCs, the markers CD105, CD73, CD90 (> 95%) and CD45, CD34 CD14, CD19 and HLA-DR (< 2%) were used; and also Adipogenic, Osteogenic, Chondrogenic Differentiation were tested.

### Intervention

Before the administration of either MSCs or placebo, history-taking and physical examination [including the Modified Medical Research Council (mMRC) dyspnea scale], routine hematological testing, inflammatory marker testing [procalcitonin (PCT), erythrocyte sedimentation rate (ESR), C-reactive protein (CRP)], chest X-ray, respiratory testing [peripheral oxygen saturation (SpO_2_), oxygenation index (PaO_2_/FiO_2_ ratio), 6 min walk (6 MW) test, peak expiratory flow (PEF)], and RT-PCR for SARS-COV-2 were performed on all subjects. All subjects received standard COVID-19 therapy according to national guidelines. In addition, NA-UC-MSCs were administered to subjects by intravenous route (50 cc MSCs suspension in 60 min using syringe pump) in the intervention group with the dose of 1 × 10^6^ cells per kilogram of body weight, as opposed to NaCl 0.9% in the placebo group.

## Outcomes

### Primary outcome

The primary outcome of this trial was the subjects' duration of hospitalization.

### Secondary outcome

The secondary outcomes of this trial were radiological and clinical indicators on baseline (day 0–2), 15 (± 2), and 22 (± 2) days: (1) Brixia score for radiographic severity; (2) mMRC Dyspnea scale; (3) oxygenation index; (4) duration of oxygen therapy (days); (5) peripheral oxygen saturation; (6) 6 MW test; (7) PEF; (8) PCT; (9) ESR; and (10) CRP levels. In addition, adverse events (AE) and serious adverse events (SAE) were recorded until day 91 (± 2) since randomization.

### Sample collection

Peripheral venous blood samples were collected for PCT, ESR, and CRP level assessment. Blood was collected on days 0–2 (baseline), 15 (± 2), and 22 (± 2) after the intervention. Following extraction, sample tubes were secured into primary (zip-lock bag), secondary (insulated bottles), and tertiary packs (insulated box with cooler packs), before being ultimately transported.

### Statistical analysis

All data in this trial were analyzed with the software SPSS V.22 (IBM). For categorical variables, analyses were computed using the χ-square or Fisher's exact test. For unpaired continuous quantities, either an independent-sample T-test (normal distribution) or an independent-sample Mann–Whitney U test (non-normal distribution) was used. In addition, the paired T-test (normal distribution) or Wilcoxon test (non-normal distribution) were performed for continuous variables.

## Conclusion

Although we have failed to demonstrate the reduction in the length of stay of severe COVID-19 patients with NA-UC-MSCs therapy, we have established that it is a very safe adjunct that yielded no AE, including serious ones, for at least 91 days after the first dose. NA-UC-MSCs therapy also did not reduce the Brixia score to values below that of the control group. However, NA-UC-MSCs improved the patients' oxygen saturation by day 22 compared to placebo, in addition to having a significantly smaller increase in PCT value compared to the control group. Thus, we are confident that MSCs-based therapies are beneficial in COVID-19 and related diseases but need fine-tuning to reach their pinnacle potential. We aim to expand this study in the near future by introducing novel delivery routes and incorporating MSCs products (i.e., secretome, extracellular vesicles (EV)).

## Supplementary Information

Supplementary Information 1.

Supplementary Information 2.

## Data Availability

The datasets generated during and/or analysed during the current study are not publicly available due to confidential data of the patients but are available from the corresponding author on reasonable request.
