# Supplementary material for: Effectiveness and safety of normoxic allogenic umbilical cord mesenchymal stem cells administered as adjunctive treatment in patients with severe COVID-19
Source: Sci Rep. 2023 Aug 2;13:12520. doi: 10.1038/s41598-023-39268-2 (PMC10397314; doi:10.1038/s41598-023-39268-2)
Supplement: Supplementary file 1 — Supplementary Information 1. [file 41598_2023_39268_MOESM1_ESM.docx]

**Supplement 1.**

The quality control of our MSCs production

| Test Method | Specification | Result of Initial Test |
| --- | --- | --- |
| Visual Attributes (subculture cells under microscope) | Small fibroblast-like spindle-cells, plastic adherent cells | Confirm |
| Visual Attributes (thawed cells in cryovial) | Clear to slightly opaque cell suspension in cryovial | Confirm |
| Adipogenic Differentiation | Positive | Positive |
| Osteogenic Differentiation | Positive | Positive |
| Chondrogenic Differentiation | Positive | Positive |
| Biomarker CD 105 | NLT 95% | 98.91% |
| Biomarker CD 73 | NLT 95% | 98.38% |
| Biomarker CD 90 | NLT 95% | 99.98% |
| Biomarker CD 45 | NMT 2% | 0.14% |
| Biomarker CD 34 | NMT 2% | 0.47% |
| Biomarker CD 14 | NMT 2% | 0.09% |
| Biomarker CD 19 | NMT 2% | 0.15% |
| Biomarker HLA-DR | NMT 2% | 0.07% |
| Cell Count | FIO | 1.275.111.111 cells |
| Viability | FIO | 96.42% |
| HIV-1 Test | Negative | Negative |
| HBV Test | Negative | Negative |
| HCV Test | Negative | Negative |
| CMV Test | Negative | Negative |
| Sterility | Sterile | Sterile |
| Karyotyping | Chromosome Normalities | Chromosome Normalities |
| Penicillin Residue | Not Detected | Not Detected |
| Mycoplasma | Negative | Negative |
| Endotoxin | <0,25 EU/mL | <0,25 EU/mL |
