# Supplementary material for: Effectiveness and safety of normoxic allogenic umbilical cord mesenchymal stem cells administered as adjunctive treatment in patients with severe COVID-19
Source: Sci Rep. 2023 Aug 2;13:12520. doi: 10.1038/s41598-023-39268-2 (PMC10397314; doi:10.1038/s41598-023-39268-2)
Supplement: Supplementary file 2 — Supplementary Information 2. [file 41598_2023_39268_MOESM2_ESM.docx]

**Supplement 2.**

The cause of death in 14 patients.

| **Caused of Early Termination (Death)** | **Subject Number** | **N** |
| --- | --- | --- |
| *Septic shock* | 01-003 | 1 |
| *Pulmonary embolism* | 02-001, 02-003 | 2 |
| *Respiratory failure* | 01-007, 01-014, 02-005 | 3 |
| *Multiple Organ Dysfunction Syndrome* | 02-004 | 1 |
| *Cardiac arrest* | 01-012 | 1 |
| *Acute respiratory distress syndrome and septic shock* | 02-010, 02-011, 03-007, 03-011 | 4 |
| *Acute respiratory distress syndrome* | 02-013, 02-014 | 2 |

*In detail of each patients*

| **Subject Number (Loc)** | **Severe Adverse Event (SAE) Description** | **In - out Hospital** | **LOS (Ds)** |
| --- | --- | --- | --- |
| 01-003 (Moewardi Hospital)  Location: SOLO | Diagnosis of SAE:   - COD direct: shock - COD indirect: sepsis   **COVID-19 confirmed cured**; HAP with septic shock with hypoxemic-hypercapnic respiratory failure, azotemia, hyperbilirubin-emia, hypoalbuminemia. The relationship between research products and SAE is unlikely. | 23 Jan 2021 – 12 Feb 2021 | 20 |
| 01-007  (Moewardi Hospital)  Location: SOLO | Diagnosis SAE:   - COD direct: respiratory failure - COD indirect: sepsis   **COVID-19 confirmed cured**; Pneumonia KR IV PSI 107 with sepsis + respiratory failure, hipoalbumin, enzim transaminase (+)  The relationship between research products and SAE is unlikely. | 8 Mar 2021 – 14 Mar 2021 | 6 |
| 01-012 (Moewardi Hospital)  Location: SOLO | Diagnosis SAE:   - COD direct: cardiac arrest - COD indirect: none   **COVID-19 confirmed cured**; the relation-ship between research products and SAE is unlikely. | 17 Jun 2021 – 29 Jun 2021 | 12 |
| 01-014 (Moewardi Hospital)  Location: SOLO | Diagnosis SAE:   - COD direct: respiratory failure - COD indirect: sepsis   COVID-19 confirmed critical degree with hypoxemic respiratory failure. The relation-ship between research products and SAE is unlikely. | 29 Jun 2021 – 06 Jul 2021 | 6 |
| 02-001 (Sardjito Hospital)  Location: JOGJA | The diagnosis of SAE is pulmonary embolism. The relationship between research products and SAE is unlikely. | 04 Feb 2021 – 19 Feb 2021 | 15 |
| 02-002 (Sardjito Hospital)  Location: JOGJA | The SAE that occurred was that the subject was admitted to the hospital on 04-08 March 2021 (the subject had previously recovered from Covid-19 and was discharged from the hospital on 02 March 2021). The diagnosis of the cause of SAE is distributive and hypo-volemic shock. The relationship between research products and SAE is unlikely. | 04 Mar 2021 – 08 Mar 2021 | 4 |
| 02-004 (Sardjito Hospital)  Location: JOGJA | The diagnosis of the cause of SAE is multiple organ dysfunction syndrome. The relationship between research products - SAE is unlikely. | 05 Mar 2021 – 15 Mar 2021 | 10 |
| 02-003 (Sardjito Hospital)  Location: JOGJA | The diagnosis of SAE is pulmonary embolism. The relationship between research products and SAE is unlikely. | 21 Feb 2021 – 18 Mar 2021 | 25 |
| 02-005 (Sardjito Hospital)  Location: JOGJA | The diagnosis of SAE is respiratory failure. The relationship between research products and SAE is unlikely. | 09 Mar 2021 – 26 Mar 2021 | 17 |
| 02-010 (Sardjito Hospital)  Location: JOGJA | The diagnosis of the cause of SAE is acute respiratory distress syndrome and septic shock. The relationship between research products and SAE is unlikely. | 21 Apr 2021 – 07 May 2021 | 16 |
| 02-011  (Sardjito Hospital)  Location: JOGJA | The diagnosis of the cause of SAE is acute respiratory distress syndrome and septic shock. The relationship between research products and SAE is unlikely. | 07 Jun 2021 – 15 Jun 2021 | 8 |
| 02-013  (Sardjito Hospital)  Location: JOGJA | The diagnosis of SAE is acute respiratory distress syndrome. The relationship between research products and SAE is unlikely. | 22 Jun 2021 – 28 Jun 2021 |  |
| 02-014  (Sardjito Hospital)  Location: JOGJA | The diagnosis of SAE is acute respiratory distress syndrome. The relationship between research products and SAE is unlikely. | 24 Jun 2021 – 05 Jul 2021 | 11 |
| 03-007  (RS Hasan Sadikin)  Location: BANDUNG | The diagnosis of the cause of SAE is acute respiratory distress syndrome and septic shock. The relationship between research products and SAE is unlikely. | 25 Jun 2021 – 07 Jul 2021 | 12 |
| 03-011  (RS Hasan Sadikin)  Location: BANDUNG | The diagnosis of SAE is Confirmed Covid-19, ARDS, and septic shock. The relationship between research products - SAE is unlikely. | 17 Jul 2021 – 02 Aug 2021 | 16 |
